# Effect of the spatial–temporal specific theca cell Cyp17 overexpression on the reproductive phenotype of the novel TC17 mouse

**DOI:** 10.1186/s12967-021-03103-x

**Published:** 2021-10-15

**Authors:** Christian Secchi, Martina Belli, Tracy N. H. Harrison, Joseph Swift, CheMyong Ko, Antoni J. Duleba, Dwayne Stupack, R. Jeffrey Chang, Shunichi Shimasaki

**Affiliations:** 1 Department of Obstetrics, Gynecology and Reproductive Sciences, School of Medicine, University of California San Diego, La Jolla, CA USA; 2 The Salk Institute for Biological Studies, La Jolla, CA USA; 3 Department of Comparative Biosciences, College of Veterinary Medicine, University of Illinois at Urbana-Champaign, Urbana, IL USA

**Keywords:** CYP17, Theca cells, Androgen excess, Mouse model, Ovary, Transgender, Fertility

## Abstract

**Background:**

In the ovarian follicle, the Theca Cells (TCs) have two main functions: preserving morphological integrity and, importantly, secreting steroid androgen hormones. TCs express the essential enzyme 17α-hydroxylase/17,20-desmolase (CYP17), which permits the conversion of pregnenolone and progesterone into androgens. Dysregulation of CYP17 enzyme activity due to an intrinsic ovarian defect is hypothesized to be a cause of hyperandrogenism in women. Androgen excess is observed in women with polycystic ovary syndrome (PCOS) resulting from excess endogenous androgen production, and in transgender males undergoing exogenous testosterone therapy after female sex assignment at birth. However, the molecular and morphological effects of *Cyp17* overexpression and androgen excess on folliculogenesis is unknown.

**Methods:**

In this work, seeking a comprehensive profiling of the local outcomes of the androgen excess in the ovary, we generated a transgenic mouse model (TC17) with doxycycline (Dox)-induced *Cyp17* overexpression in a local and temporal manner. TC17 mice were obtained by a combination of the Tet-dependent expression system and the Cre/LoxP gene control system.

**Results:**

Ovaries of Dox-treated TC17 mice overexpressed *Cyp17* specifically in TCs, inducing high testosterone levels. Surprisingly, TC17 ovarian morphology resembled the human ovarian features of testosterone-treated transgender men (partially impaired folliculogenesis, hypertrophic or luteinized stromal cells, atretic follicles, and collapsed clusters). We additionally assessed TC17 fertility denoting a perturbation of the normal reproductive functions (e.g., low pregnancy rate and numbers of pups per litter). Finally, RNAseq analysis permitted us to identify dysregulated genes (*Lhcgr*, *Fshr*, *Runx1*) and pathways (Extra Cellular Matrix and Steroid Synthesis).

**Conclusions:**

Our novel mouse model is a versatile tool to provide innovative insights into study the effects of *Cyp17* overexpression and hyperandrogenism in the ovary.

**Supplementary Information:**

The online version contains supplementary material available at 10.1186/s12967-021-03103-x.

## Introduction

Ovarian follicles are comprised of three diverse cell populations: oocytes, granulosa cells (GCs), and theca cells (TCs) [1–5]. TCs comprise the outer portion of the follicle (3–5 layers) and have two main functions: preserving the morphological integrity of follicles and, importantly, the production of androgen steroids [6, 7]. These functional roles of the theca layer are mainly achieved by two different cell types, an inner theca interna and an outer theca externa [8]. The terminal differentiation of the theca externa is characterized by expression of contractile proteins that play critical roles leading to follicle rupture in the ovulation process [9]. On the other hand, terminal differentiation of the theca interna is characterized by development of theca interstitial cells that produce androgens. Understanding this developmental process is important because theca-derived androgens are obligatory precursors for follicle estrogen production, and are essential for normal folliculogenesis, ovulation, and reproduction [10].

Focusing on TCs and their androgenic role in female reproduction has been conceptually overlooked, with androgens being primarily studied in the context of male reproductive function. However, in recent decades, novel cellular and molecular methodologies as well as animal models have revealed new insights into the impact of TC androgen production on follicle progression and the onset of ovarian disorders such as polycystic ovary syndrome (PCOS) [11–18]. Therefore, understanding the role of TCs and their androgenic steroids on follicle maturation has significantly evolved over time [19]. Initially, androgens were considered detrimental to normal folliculogenesis, mainly due to the negative effects demonstrated in mouse models [20–23]. The afterward evolving narrative, built on androgen receptor (AR) investigations [24, 25], appeared to be more complex. Indeed, TCs were found to also promote crosstalk among GCs and oocytes during development and to support the growing follicle as it progresses through developmental stages to produce a mature oocyte [26–28]. In vitro studies have shown that androgen can promote GC differentiation, but this appeared to be stage dependent as with increased growth, larger size follicles may become less responsive and even atretic in response to androgen exposure [13, 17, 29]. To date, it is known that androgens are important for normal ovarian follicle maturation since small follicle growth is enhanced by androgens [13, 17, 29–31]. It has been hypothesized that this effect might be the result of the ability of androgens to increase FSH receptor in GCs [14, 15].

Notably, steroidogenic TCs uniquely express the essential enzyme 17α-hydroxylase/17,20-desmolase (CYP17), which is required for androgen production [7, 32–34]. In the female mouse, *Cyp17* expression is primarily restricted to the ovary (~ 500 transcripts per million, TPM) and placenta, with faint expression (~ 2 TPM) in the uterus and adrenals. Within ovarian follicles, *Cyp17* is expressed in TCs but not in adjacent GCs or in oocytes [35, 36]. Most importantly in women with PCOS, androgen overproduction likely results, at least in part, from dysregulation of Cyp17 enzyme activity due to an intrinsic defect of the TCs [37–39]. This is supported by studies demonstrating elevated levels of *Cyp17* mRNA and protein expression in TCs of ovaries from women with this disorder [30, 40]. However, most of these studies were performed in PCOS patients and, therefore, are associated with intrinsic morphological and functional ovarian defects that cannot recapitulate the genuine role of TCs in the normal ovary. Therefore, the physiological role of androgens on follicle function remains unclear. This limitation is not trivial since comprehensive knowledge of the effects of androgens on ovarian function in normal women is very limited. The closest experimental evidence, appropriately focused on the androgens effect in non-pathological ovaries, have been transgender male (TGM) studies which were unfortunately characterized by limited power and lack conclusive results [41–43].

As a result, there is an absence of reliable information regarding the effect of androgen on normal follicle function. To address these gaps in knowledge, we created, by a combination of the Cre/LoxP and the Tet-dependent (on–off switch) expression systems, a transgenic mouse model inducibly overexpressing *Cyp17*, which we called TC17. This strategy differs from other animal models of androgen excess that have involved in vivo and systemic administration of a single androgen or aromatase inhibitor (e.g., Letrozole) [44–46]. Remarkably, our TC17 recapitulated the ovarian morphology observed in TGM treated with gender affirming testosterone therapy and appears to be a valuable model to study the ovarian folliculogenesis in presence of local long term androgen excess.

## Materials and methods

### Plasmids and mouse models

**Fig. 1:**
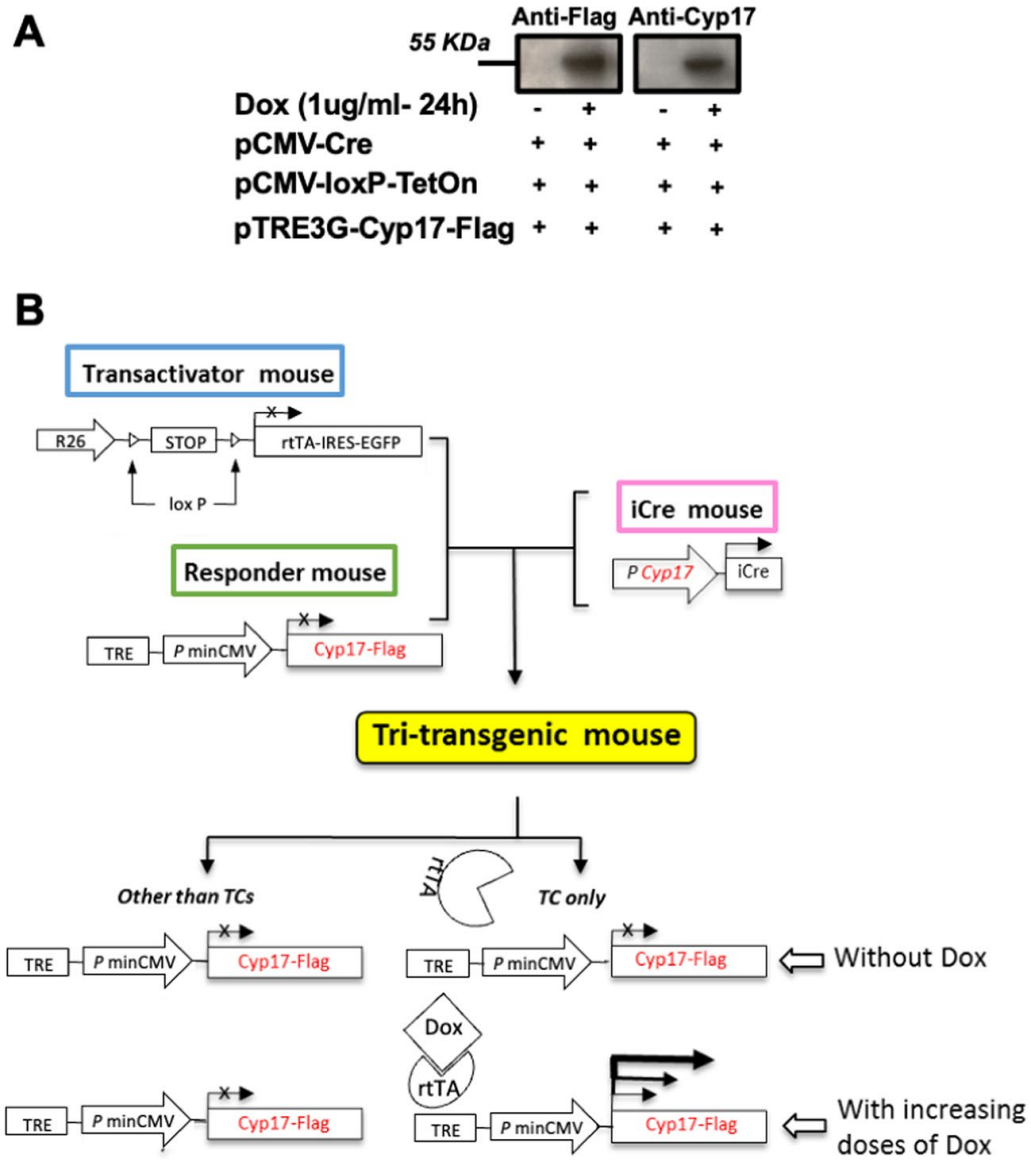
TC17 validation in vitro and in vivo strategy. **A** 293 T cells were transfected with three plasmids containing CRE, LoxP/TetOn, and Cyp17/Flag. After exposure for 24 h with or without 1 ug/ml Dox, cell lysates were subjected to immunoblotting analysis with anti-Flag and anti-Cyp17 antibodies. **B** Homozygous transactivator mice were bred with homozygous responder mice. Double transgenic mice were then bred with Cyp17iCre transgenic mice [60] to produceR26-STOP-rtTA-IRES-EGFP/TRECyp17/Cyp17iCre triple transgenic mice. R26, a ubiquitous and endogenous ROSA26 promoter; rtTA, reverse Tet-controlled transcriptional activator; IRES, internal ribosome entry site; EGFP, enhanced green fluorescent protein; Dox, doxycycline

All mice were C57BL/6 J (B6) background (Jackson or Envigo, USA). We generated a breeding line of mice overexpressing TC-selective *Cyp17* using a combination of the Tet-dependent expression system and the Cre/LoxP gene control system as outlined in Fig. 1B. The combination of Tet-based induction and Cre/LoxP gene control is a newer system developed to produce transgenic animal models to study the molecular basis of human disease in adult animals in a temporal manner. This elegant strategy is widely used in vivo and in vitro for conditional, reversible gene expression [47–59]. Specifically, we have used *Cyp17* promoter-iCre mice [60] crossed with trans-activator mice (R26-STOP-rtTA-IRES-EGFP transgene at the ROSA26 locus, Jackson Lab) and with responder mice carrying the TRE-Cyp17 transgene created at the University of California, San Diego (UCSD) transgenic mouse and embryonic stem cell core facility. The *Cyp17* coding segment was inserted into the multi-cloning site of the TRE-PminCMV vector (pTRE-TightTM, Clontech, Mountain View, CA) which contains a modified Tet responsive element (TRE) that is silent in the absence of rtTA and Doxycycline (Dox) treatment. A two-step breeding process was used to obtain transgenic mice. Initially, we paired transactivator and responder mice to produce double transgenic mice (R26-STOP-rtTA-IRES-EGFP/TRE-Cyp17). Subsequently, we mated the double transgenic mice with iCre-expressing mice to get the experimental tri-transgenic mice (R26-STOP-rtTA-IRES-EGFP/TRE-Cyp17/Cyp17iCre). Here, *Cyp17* promoter-iCre mice [60] were used to ensure rtTA/EGFP was expressed specifically in TCs of secondary follicles. Importantly, after the DNA segment between the two LoxP sites was excised by Cyp17iCre specifically in TCs, the R26-STOP-rtTA gene remained activated in all daughter TCs. Double transgenic mice with WT *Cyp17* gene (R26-STOP-rtTA-IRES-EGFP/Cyp17iCre) and double transgenic mice without rtTA/TetOn gene (TRE-Cyp17/Cyp17iCre were considered control mice (CTRL) for the study. Only upon treatment with Dox can suppression be relieved and active transcription of TRE-Cyp17 be induced. Pilot studies were performed to validate the model (Fig. 1A, Additional file 1: Figure S1, Additional file 1: Figure S2). Progressive administration of intraperitoneal (i.p.) injected Dox exerted a dose-dependent induced gene expression increase in vivo in transgenic mice (Additional file 1: Figure S1). A pilot in vitro study was performed to validate the efficiency of the systems (Fig. 1A). Mice were group-housed in a temperature-controlled room (21–22 °C) with a 12-h light/dark cycle and ad libitum access to food and water. All animal experiments were carried out in accordance with the Institutional Animal Care and Use Committee-approved protocol (#S01022) at UCSD. The sample size and age of the animals used are indicated in the figure legends. To validate effective long-term upregulation of *Cyp17*, we used a commercially available, higher dose Doxycycline hyclate diet as a more convenient administration method (TD.120489 2020, 994 g/Kg Teklad Global Soy Protein-Free Rodent Diet, pre-extruded and 6 g/Kg Doxycycline Hyclate), which contains approximately 87% doxycycline. This diet was designed by the vendor to deliver a daily dose of 16–26 mg of Dox based on consumption of 3–5 g/d by a mouse. The standard control diet (TD.140163 2020, Teklad Global Soy Protein-Free Rodent Diet, pre-extruded) was given to all mice during breeding, lactation, and growth of the young stock. Mice were randomly assigned to groups at the time of weaning to minimize any potential bias. Health status was normal for all animals at the beginning of the experiments.

### Tissue collection and histology

After Dox chow exposure, mice were anesthetized with isoflurane, weighed, and blood was collected via retro-orbital bleeding before rapid euthanizing. After extraction, ovaries were weighed. One ovary from each mouse was collected, frozen on dry ice, and stored at − 80 °C until processing for mRNA expression levels using quantitative PCR. The other ovary from each mouse was fixed in 10% formalin at 4 °C overnight and stored in 70% ethanol before histologic processing.

For histological analysis, fixed ovaries were serially sectioned at 10 μm and then stained with hematoxylin and eosin (H&E) by the UCSD Tissue Technology Shared Resource (formerly known as Histology and Immunohistochemistry Core). Primary, secondary, antral, and cystic follicles were counted from two sections randomly selected from each ovary. In each case, counts were made by one investigator blind to the treatment group.

### Hormone assays

LH, FSH, and E2 hormone levels were measured by the University of Virginia Ligand Core Facility. Serum LH and FSH were measured by a mouse multiplex assay (reportable range 0.24–30.0 ng/ml and 2.4–300 ng/ml, respectively). Serum E2 was measured using a mouse enzyme-linked immunosorbent assay (range 3.0–300 pg/ml). Serum T was measured with LC–MS/MS at the UCSD Health Center for Advanced Laboratory Medicine (range 4–1560 ng/dL).

### Estrous cycle and fertility assessment

Estrous cycles were monitored over a period of 15 days by light microscopic analysis of the predominant cell type in vaginal epithelial smears obtained 4–6 weeks after Dox or control chow treatment. Proestrus was categorized by the presence of nucleated and some cornified epithelial cells, estrus by the presence of cornified cells, and metestrus/diestrus for the presence of some cornified epithelial cells and primarily leukocytes. A separate cohort of female TC17 and CTRL mice at 7 weeks old was used to assess fertility. At the age of 8 weeks and 1 week after treatment (n = 10/group), TC17 were paired with adult C57BL/6 N breeder males at 3 months old. Breeder males were removed after 10 days, and females were assessed for pregnancy, time to first litter, and number of pups per litter.

### Hematological profile

After collection of blood, Hemavet 950FS was used to obtain the hematological profile of TC17 and CTRL mice. The control ranges for RBC (M/μl) and for HCT (%) were 6.36–9.42 and 35.1–45.4, respectively.

### Transient transfections

293 T cells were cultured into 6-well tissue culture plates (1 × 10^6^ cells/well). The medium was replaced the day after with serum free DMEM-F12 (complemented with antibiotics), and transfection was performed using the above-described plasmids for 4 h with Lipofectamine LTX with Plus or Lipofectamine 3000 reagents (catalog #15338100 or #L3000008, respectively, Thermo Fisher Scientific) following manufacturer’s procedures. After 4 h, the medium was replaced with new serum free DMEM-F12 and transfected cells were cultured at 37 °C for 24 h with or without Dox. At this point, cells were lysed for further analyses.

### RNA extractions and q-RT-PCR

After 4-h transfection and additional culture for 24 h in serum free DMEM-F12, 293 T and total ovaries were lysed using TRIzol reagent (catalog #15596026, Thermo Fisher Scientific) and RNA was extracted with Direct-zol RNA MiniPrep kit (#R2052, Zymo Research, Irvine, CA) following manufacturer’s protocol. High-Capacity cDNA Reverse Transcription Kit (#4368814, Thermo Fisher Scientific) was used to reverse transcribe 1 μg RNA. mRNA expression was quantified by q-RT-PCR amplification of cDNA using SYBR Green PCR Master Mix (#4309155, Thermo Fisher Scientific) and a Bio-Rad CFX384 Real-Time PCR Detection System. Q-RT-PCR was performed with primer assays from Qiagen RT^2^ qPCR Primer Assay (200) (catalog #330001) targeting the *Cyp17, Cyp19*, *Pgr*, *Ahm*, *Lhcgr, Fshr* and, *Foxl2* gene. Primer assay efficiencies were guaranteed by the manufacturer. Target gene expression was normalized on *Gapdh* and *Actin* expression.

### RNAseq analysis

Total TC17 ovaries were smashed and lysed using TRIzol reagent (catalog #15596026, Thermo Fisher Scientific) and RNA was extracted with Direct-zol RNA MiniPrep kit (#R2052, Zymo Research, Irvine, CA) following manufacturer’s protocol. RNAseq was performed by Novogene Inc. Briefly, mRNA was purified from total RNA using poly-T oligo-attached magnetic beads. Fragmentation was carried out using divalent cations under elevated temperature in NEBNext First Strand Synthesis Reaction Buffer (5×). First strand cDNA was synthesized using random hexamer primer and M-MuLV Reverse Transcriptase (RNase H). Second strand cDNA synthesis was subsequently performed using DNA Polymerase I and RNase H. Remaining overhangs were converted into blunt ends via exonuclease/polymerase activities. After adenylation of 3′ ends of DNA fragments, NEBNext Adaptor with hairpin loop structure was ligated to prepare for hybridization. In order to select cDNA fragments of preferentially 150–200 bp in length, the library fragments were purified with the AMPure XP system (Beckman Coulter, Beverly, USA). Then 3 µl USER Enzyme (NEB, USA) was used with size-selected, adaptor ligated cDNA at 37 °C for 15 min followed by 5 min at 95 °C before PCR. Then PCR was performed with Phusion High-Fidelity DNA polymerase, Universal PCR primers and Index (X) Primer. At last, PCR products were purified (AMPure XP system) and library quality was assessed on the Agilent Bioanalyzer 2100 system. Sequencing libraries were generated using NEBNext UltraTM RNA Library Prep Kit for Illumina (NEB, USA) following the manufacturer’s recommendations and index codes were added to attribute sequences to each sample. RNA-seq libraries were sequenced on the Novaseq4000, with 150 bp paired end read chemistry. Reads were aligned to the Mus musculus genome using STAR (mismatch = 2). Read quantification was called using FeatureCounts, and differential expression analysis was called using DESeq2, with an adjusted p-value threshold of 0.05. Additionally, GO and KEGG enrichment analyses were performed using ClusterProfiler using an adjusted p-value threshold of 0.05 [61–63].

### Confocal and RNAscope

Mice were deeply anesthetized with Medetomidine/Ketamine. When no response to tail/toe pinch was present, mice were transcardially perfused with 1% phosphate-buffered saline solution first, followed by 4% paraformaldehyde solution to fix the ovary tissue. The ovaries were then removed from the mice and kept in a 30% sucrose solution until use. Frozen ovaries were sectioned (30 μm) with a standard Leica Cryostat (CM1860). Fluorescent images of four ovary sections per mouse were acquired with a Leica Confocal microscope. RNAscope in situ hybridization (ACD, Advanced Cell Diagnostics) for murine *Cyp17* mRNA was performed following manufacturer instructions. Based on the design of ACD double ZZ probe pairs, both need to bind to the target to begin amplification, any mismatched targets are ignored prior to amplification and therefore the obtained signal is extremely specific. The probe for *Cyp17* (ACD Ref. 522611, GenBank Accession Number NM_007809.3) was designed to target nucleotides 159–1100 of the cDNA sequence. RNAscope^®^ 3-plex Negative Control Probe (ACD Ref. 320871, bacterial gene dapB of Bacillus subtilis strain) was used to assess any nonspecific background.

### Western blot analysis

After 4-h transfection and additional culture for 24 h in serum free DMEM-F12, HGrC1 cells were lysed in lysis buffer (RIPA buffer, catalog #89901, Thermo Fisher Scientific), phosphatase inhibitor cocktail (#78420, Thermo Fisher Scientific) and protease inhibitor cocktail (#P8340, Sigma-Aldrich). Pierce BCA protein assay kit was used for total protein quantification (#23227, Thermo Fisher Scientific). NuPAGE LDS sample buffer 4× (#NP0007, Thermo Fisher Scientific) and β-mercaptoethanol (#6010, Calbiochem, Billerica, MA) were added to cell lysates, and samples were denaturized at 95 °C for 5 min. Protein separation occurred on 12% SDS-PAGE gels and with subsequent transfer to nitrocellulose membranes. Membranes were then incubated for 1 h with blocking solution (BSA, #A30075-100, Research Products International, Mount Prospect, IL), and with primary antibodies overnight at 4 °C. Membranes were washed three times, then incubated for 1 h with horseradish peroxidase-conjugated secondary antibodies, further washed three more times and incubated with Super Signal West Femto Maximum sensitivity substrate to detect chemiluminescence (#34095, Thermo Fisher Scientific). Alternatively, primary (antibody Proteintech cat. #14447-1-AP for Cyp17A1, antibody Sigma cat.#F1804 against FLAG tag) and secondary antibody incubations were performed using solutions 1 and 2, respectively, of Signal Enhancer HIKARI, (#NU00101 and #NU00102, Nacalai USA, San Diego, CA) to enhance protein detection. β-actin was used as a loading control.

### Statistic analysis

Sample sizes were selected based upon our experience with the assays being performed to achieve sufficient power to detect biologically relevant differences in the experiments being conducted. Unpaired *t*-test, unpaired *t*-test with Welch’s correction, Mann–Whitney test, and were performed where appropriate as reported in the figure legends. All statistical analyses were performed using GraphPad Prism software. A comparison was considered significant if P was less than 0.05.

## Results

### Dox treatment in TC17 transgenic mice induced expression of *Cyp17* in a local and temporal manner and increased Testosterone blood levels

After in vitro validation of the trans genetic constructs used in the present work (Fig. 1A) and the execution of breeding strategy to obtain TC17 mice (see Material and Methods and Fig. 1B), the novel TC17 model was dox-treated by *i.p.* injection for a dose–response (Additional file 1: Figure S1).

**Fig. 2:**
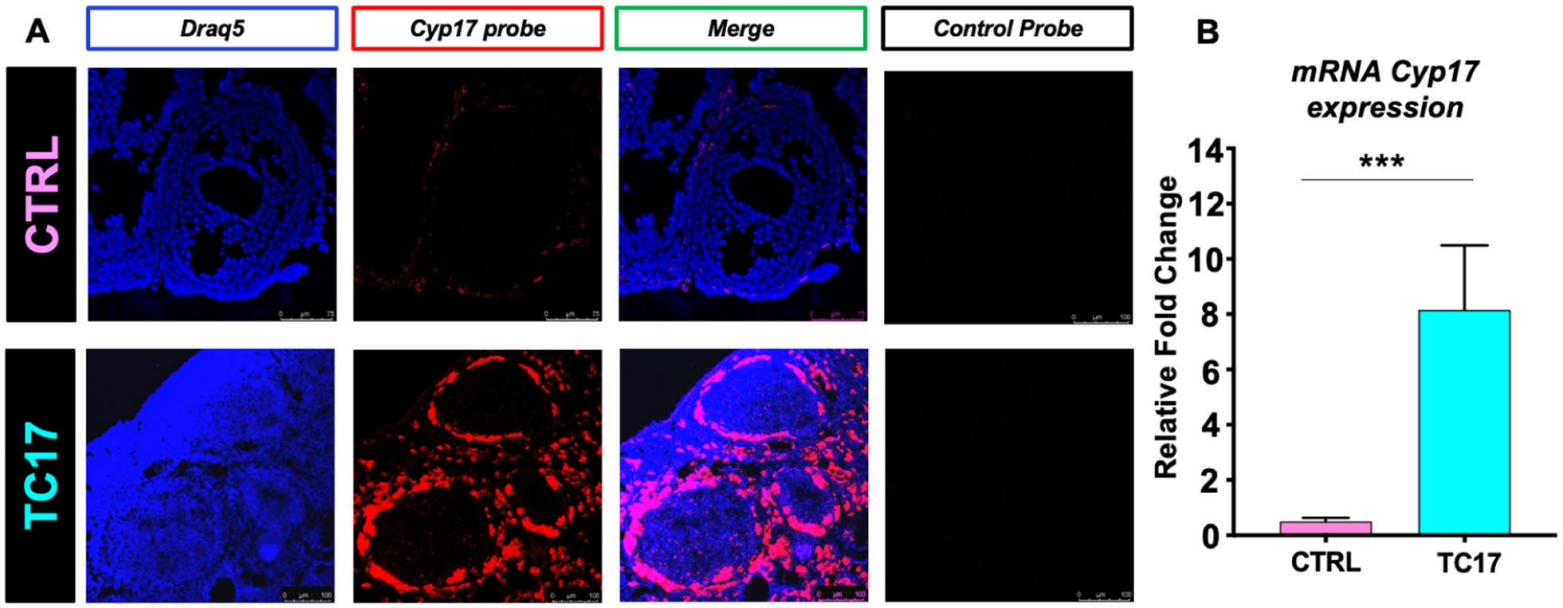
TC17 validation in vivo. Treatment started at 8 weeks old mice. **A** After Dox treatment (4 weeks), CTRL and TC17 mice were sacrificed, and ovaries were collected (N = 4). RNAscope was performed for *Cyp17* probe and Draq5 to stain DNA. Representative confocal micrographs of CTRL (upper panel) and TC17 transgenic mouse ovaries (lower panel). Panels show the effects of Dox treatment in the Cyp17 expression (in red). An antisense control probe was used as control from the vendor. **B** qPCR validation of *Cyp17* expression in TC17 ovaries compared with CTRL ovaries (N = 6). After Dox treatment (8 weeks), ovaries were collected, and qPCR was performed Graphs show fold change means ± s.e.m relative expression to *Cyp17* following normalization to the housekeeping gene. Data were analyzed using the two-tailed Mann–Whitney test (***p < 0.001)

The expression of *Cyp17* appeared to be dose dependent. To validate the effective long-term upregulation of *Cyp17*, we used Dox diet as a more convenient administration method. After long-term treatment (4 weeks) mice were euthanized, and *Cyp17* upregulation was validated by RNAscope and qPCR (Fig. 2). Strikingly, RNAscope analysis of the TC17 ovaries clearly showed specific upregulation of *Cyp17* in TCs compared with the CTRL (Fig. 2A). Increased *Cyp17* mRNA levels were analyzed and confirmed by qPCR (Fig. 2B). Also, the system specifically expressed the transactivator rtTA protein in the TC17 TCs as shown by microscopy (Additional file 1: Fig. 2).

**Fig. 3:**
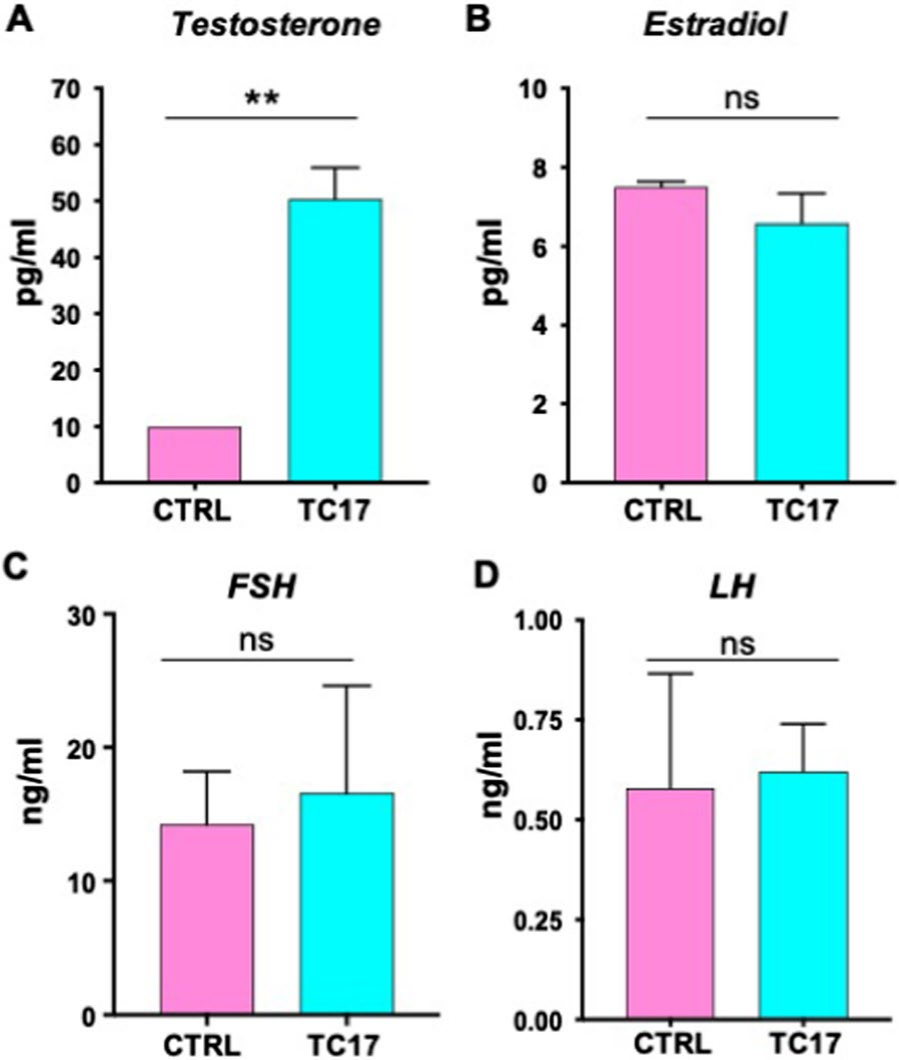
Endocrine profile in TC17 female mice. After Dox treatment (8 weeks) TC17 and CTRL blood sera (N = 6) were collected, and hormone levels were quantified. Means ± s.e.m serum levels of T (**A**), E2 (**B**), FSH (**C**), and LH (**D**) in TC17 and CTRL females. Data were analyzed using the two-tailed Mann–Whitney test (**p < 0.01)

To further validate our model, we quantified hormone levels in the serum. As expected, T was strongly upregulated in TC17 compared to CTRL (Fig. 3A). E2, FSH, and LH did not show significant differences (Fig. 3B–D).

### TC17 ovarian phenotype was marked by impaired folliculogenesis, hypertrophic luteinized stomal cells, follicle atresia, and collapsed cell clusters

**Fig. 4:**
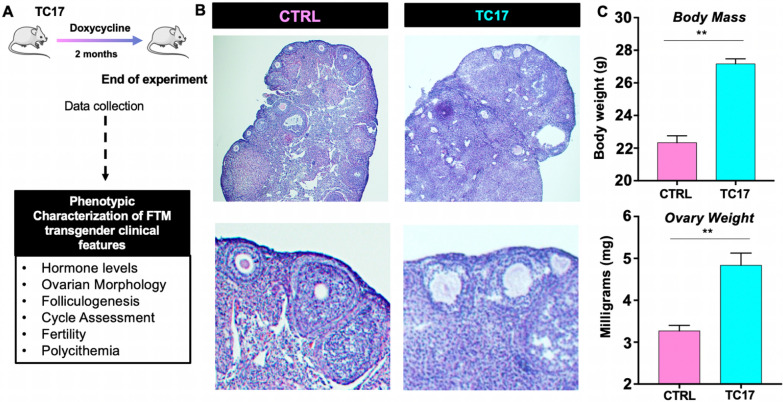
TC17 ovarian morphology demonstrates altered folliculogenesis. **A** General scheme of a long-term study in TC17. After Dox treatment (8 weeks), CTRL and TC17 mice were sacrificed, and ovaries were collected (N = 6). **B** Histological analysis of ovaries stained with H&E at the end of the treatment course. Top panels, representative images. Bottom panels, insets of images in top panels. **C** Body mass (grams, top panel) and ovarian weight (mg, bottom panel) in CTRL and TC17 mice (N = 6), means ± s.e.m. Data were analyzed using the two-tailed Mann–Whitney test (**p < 0.01)

To further understand the long-term effect of the induced *Cyp17* upregulation, female TC17 mice were treated with Dox for 8 weeks (Fig. 4). The schematic execution of the experiments is depicted in Fig. 4A. After treatment, TC17 body mass and ovarian weight were significantly higher compared to the CTRL mice (Fig. 4C). Histological assessment of the ovaries showed that TC17 presented a different morphology compared with the control with a high presence of stromal cells (Fig. 4B).

**Fig. 5:**
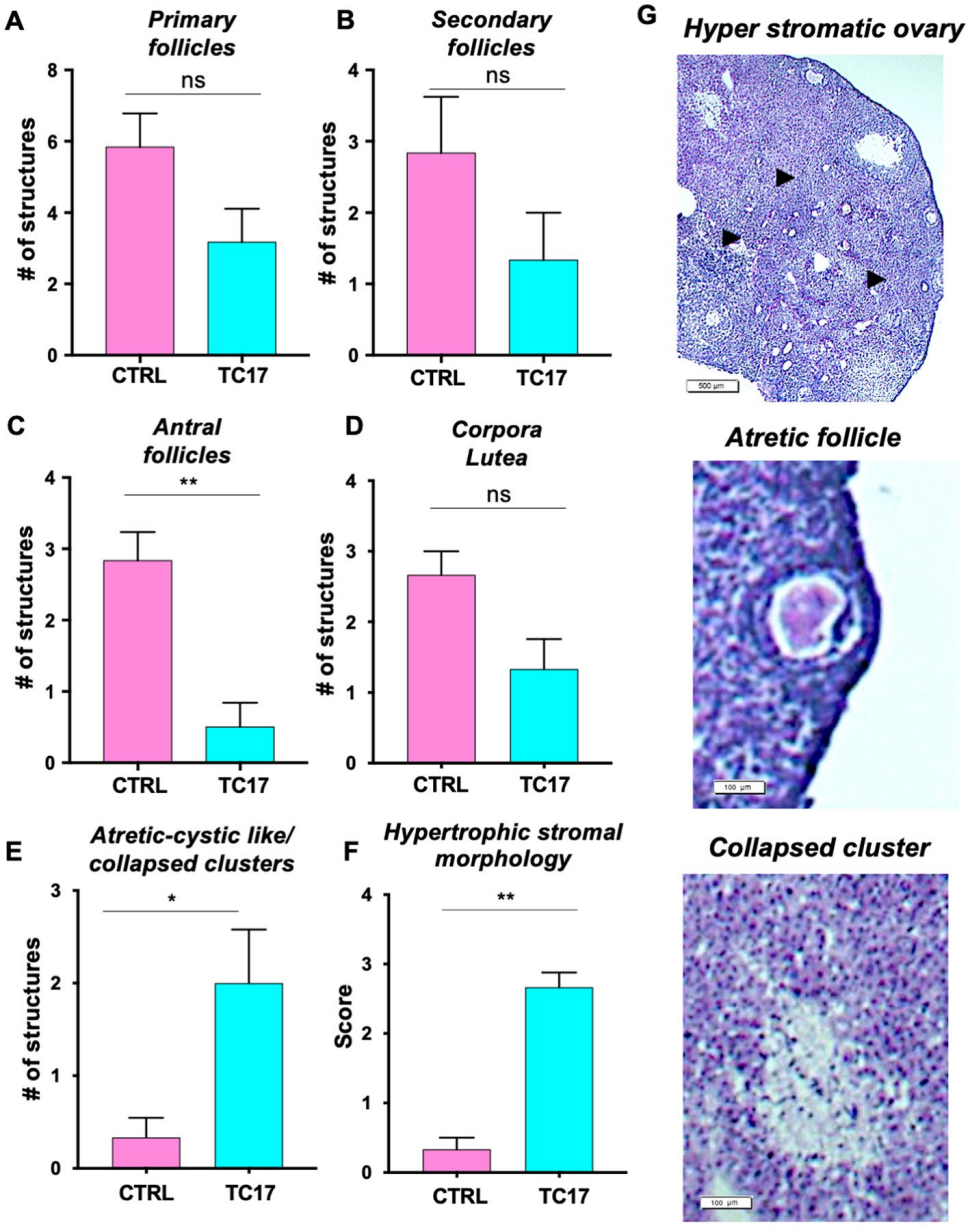
Follicular morphology assessment of TC17 ovaries. After Dox treatment (8 weeks), CTRL and TC17 mice were sacrificed, and ovaries were collected, and histological assessment was performed (N = 6). **A**–**E** Primary follicles (**A**), secondary follicles (**B**), antral follicles (**C**), corpora lutea (**D**) and atretic-cystic/collapsed clusters (**E**) were quantified in TC17 and CTRL mice, means ± s.e.m. **F** Histological scores of hypertrophic stromal morphologies stained with H&E at the end of the treatment (0–1 = normal; 2 = mild hypertrophy; 3 = severe hypertrophy), means ± s.e.m. Data were analyzed using the two-tailed Mann–Whitney test (*p < 0.05, **p < 0.01). **G** Representative images of hyperstromatic follicles (top panel), an atretic follicle (middle panel) and a collapsed cluster (bottom panel)

TC17 ovaries were also characterized by impaired folliculogenesis with a significantly lower number of antral follicles (Fig. 5A–D and G) and hypertrophic stromal or luteinized stromal cells (Fig. 5F and G). Moreover, atretic follicles and atretic cystic formations were observed (Fig. 5E). We also found morphological structures that relate to the luteinization of stroma rather than corpora. These structures (Fig. 5G)—which we called collapsed clusters—were composed of pools of lucent cells not discernable from the surrounding stroma.

### TC17 female mice have delayed estrous cycle and impaired fertility

**Fig. 6:**
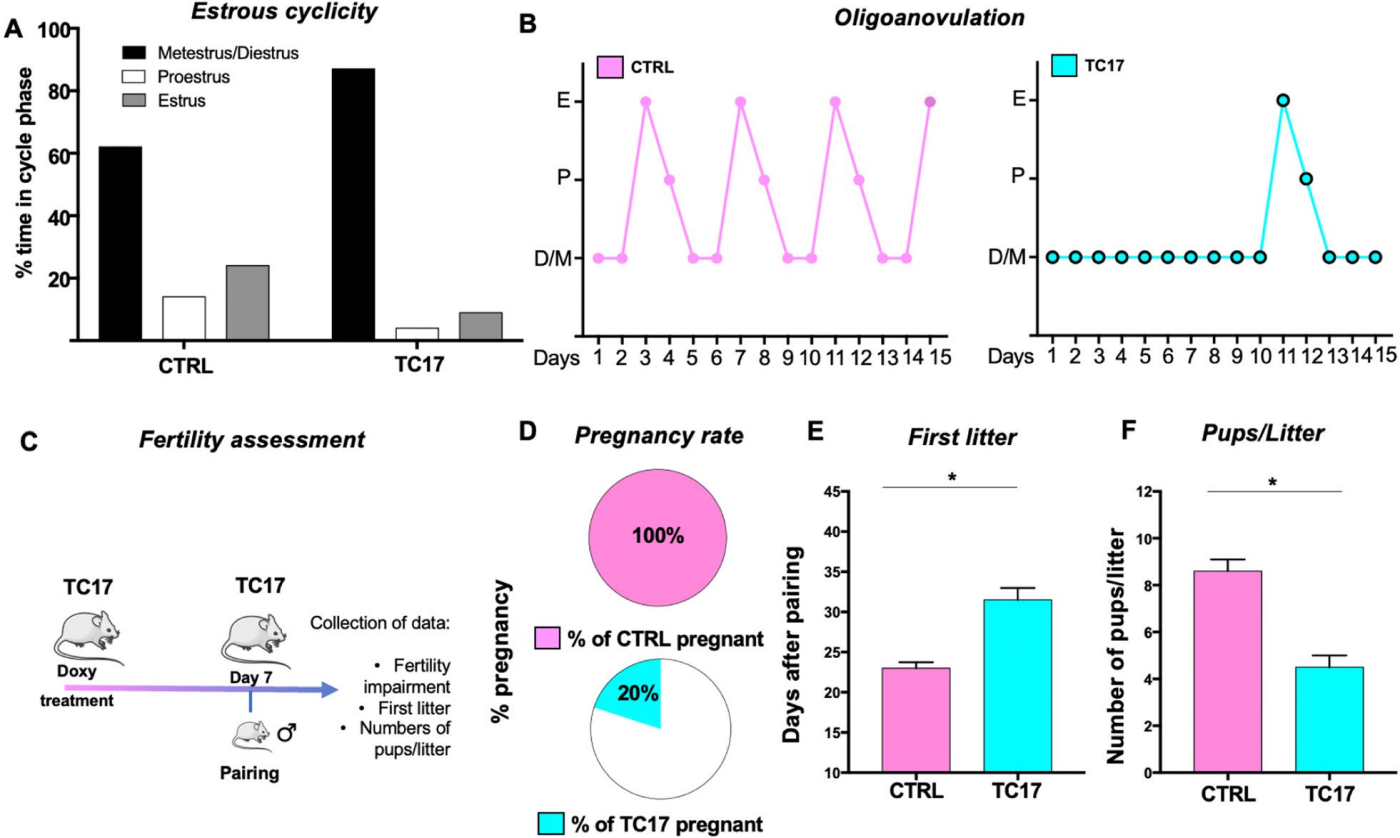
Estrous cycle and fertility assessment in TC17 mice. During the Dox treatment (8 weeks), the estrous cycle in CTRL and TC17 mice (N = 10) was assessed at 4 weeks after the start of the treatment. Proestrus was categorized by the presence of nucleated and some cornified epithelial cells, estrus by the presence of cornified cells, and metestrus/diestrus for the presence of some cornified epithelial cells and primarily leukocytes. **A** Percentage of the relative amount of time spent in each cycle stage, **B** Representative estrous cyclicity of six mice/group during 15 consecutive days. M/D, metestrus/diestrus phase; P, proestrus; E, estrus. **C** Schematic graphic of the experimental plan for the A separate cohort of female mice of TC17 and CTRL mice at 7-week-old was used to assess fertility. At 8 weeks of age, 1 week after treatment (N = 10) were paired with adult C57BL/6 N breeder males (3-months-old). Breeder males were removed after 10 days. Females were assessed for pregnancy rate (**D**), time (days) to first litter (**E**), and a number of pups/litter, means ± s.e.m. Data were analyzed using the two-tailed Mann–Whitney test (*p < 0.05)

The evidence that TC17 ovaries can resemble that observed in transgender men creates a potential model for studying the effects of long-term hyperandrogenemia on the reproductive physiology of these individuals. As the first approach, we assessed the TC17 estrous cycle. At 5 weeks after treatment, TC17 female mice spent their estrous cycle in diestrus, according to the finding that leukocytes were predominant in their vaginal smears (Fig. 6A) and presented partial oligo-anovulation with an evident delay of the estrous (Fig. 6B). To assess functional consequences of the upregulation of *Cyp17* and ovarian hyperandrogenism on reproduction, we evaluated fertility in TC17 and CTRL females beginning 1 week after Dox treatment (scheme of the experiment depicted in Fig. 6C). All the CTRL females (10/10, 100%) gave birth to a litter following pairing with a breeder male for 10 days (Fig. 6D). In contrast, only two of the TC17 females (2/10, 20%) successfully gave birth. Signs of impaired fertility were also evident by significant differences in the time to birth the first litter (Fig. 6E) and litter size (Fig. 6F) compared to the CTRL group.

### Transcriptional profiling of TC17 ovaries reveals genome-wide impact of Cyp17 upregulation

**Fig. 7:**
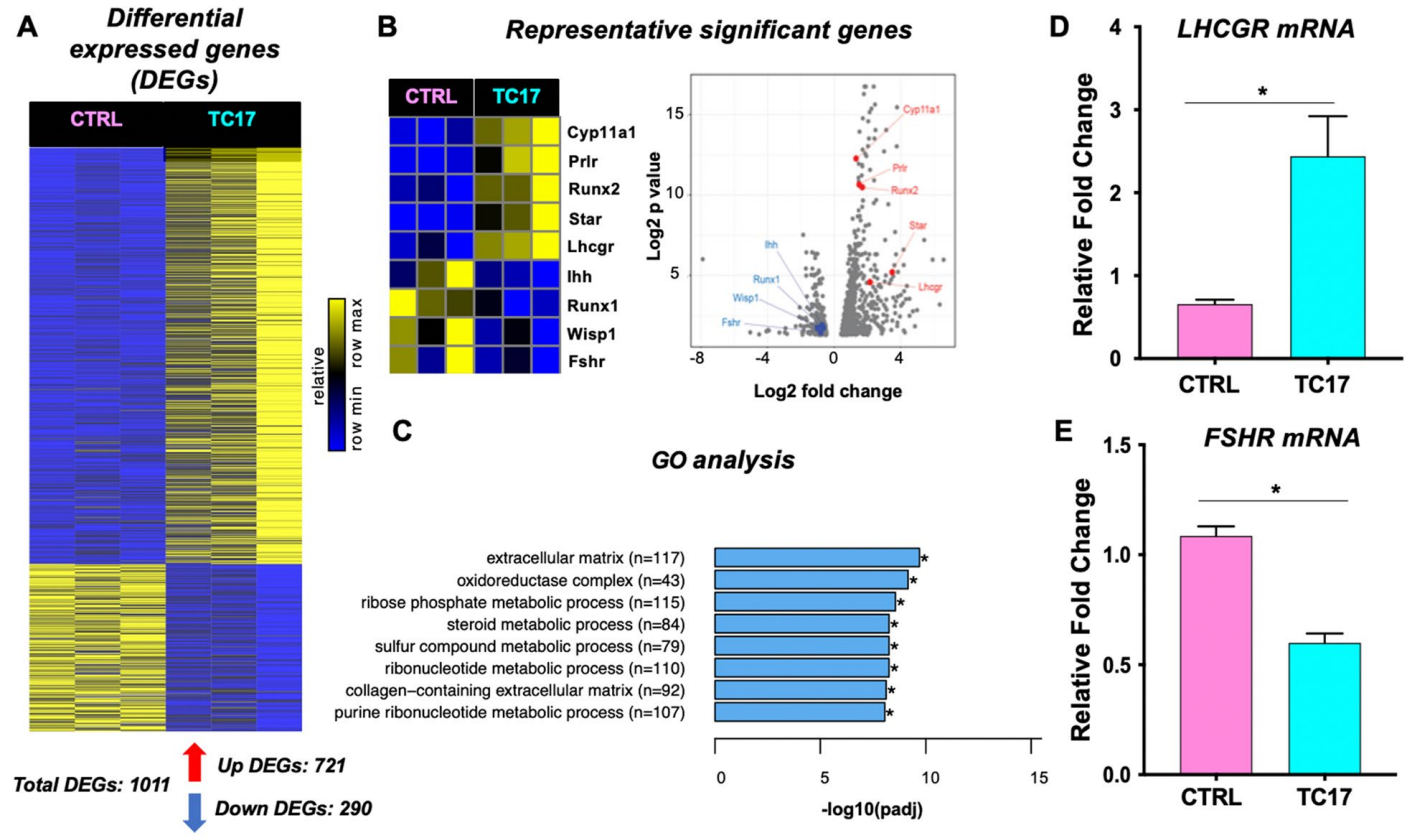
The transcriptomic effect of Cyp17 overexpression is the TC17 ovary. RNA sequencing reveals *Cyp17* effect on ovary gene regulation. **A** Upon *Cyp17* induction, 721 genes were upregulated, and 290 genes were downregulated (adj. p-value < 0.05). **B** Among these genes, well-known ovary morphogenesis transcriptional regulators such as *Cyp11, Prlr* were upregulated, while *Ihh* and *Runx1* were downregulated. Volcano plot indicates the relative fold change and p-value of genes in **B**. **C** GO analysis of the 1011 differentially expressed genes included terms such as extracellular matrix and collagen-containing extracellular matrix (see Additional file 1: Figure S4 for full-term list). **D**–**E** The induction and repression of *Lhcgr* and *Fshr* respectively, as found by RNA-seq, were confirmed using qPCR

To investigate the transcriptional changes associated with the Dox-induced *Cyp17* upregulation, and thus the effect of excess androgen, TC17 and CTRL ovaries were profiled using RNA sequencing (Fig. 7A). We then performed a differential expression analysis between CTRL and TC17 samples to identify genes induces or repressed by *Cyp17* overexpression (Additional file 2: Table S1).

We found a total of 1011 differentially expressed genes (DEGs) of which 721 were upregulated, and 290 were downregulated (Fig. 7A, bottom). The heatmap analysis and volcano plot in Fig. 7B shows upregulation (e.g.,* Prlr*, *Cyp11a*, *Star*, *Runx2*) or downreguation (e.g., *Fshr*, *Runx1*) of genes known to be integral to ovary morphogenesis. Additional file 1: Figure S3 shows the 50-top down- and upregulated DEGs (ranked by adjusted p-value). We independently verified *Lhcgr* and *Fshr* mRNA levels by qPCR (Fig. 7D and E). Other important ovarian markers (*Cyp19*, *Pgr*, *Amh*, *Foxl2*) were validated in the same manner (Additional file 1: Figure S6).

Next, to evaluate the functional significance of the genes found to be dysregulated in the TC17 model, we performed Gene Ontology (GO) analysis on DEGs. We found DEGs (Additional file 1: Figure S4) were significantly enriched in the extracellular matrix (ECM), collagen-containing ECM, and steroid metabolic process pathways (Fig. 7C, Additional file 1: Figure S5). These findings can help explain our histopathological findings, in which the main features were an increase of the stromatic component/luteinized tissue (see an increase of LH receptor and enriched GO Pathways associated with collagens and ECM) with specific transgender features and a partial impairment of the folliculogenesis (decreased *Fshr* levels).

### TC17 mice have polycythemia

Finally, we sought to investigate if TC17 presented specific systemic signs associated with androgen overload. We found that TC17 blood was distinguished by polycythemia, with elevated Red Blood Cell (RBC) levels and hematocrit (HCT) percentage, as depicted in Additional file 1: Figure S7.

## Discussion

In this work, we present a new transgenic mouse model, called TC17, which is characterized by the Dox-induced spatial and temporal *Cyp17* upregulation in TCs. We generated responder mice with pTRE3G-Cyp17. By crossing these mice with transactivator mice (R26-STOP-rtTA-IRES-EGFP transgene at the ROSA26 locus) and iCre mice (*Cyp17* promoter-iCre), we successfully obtained tri-transgenic mice overexpressing Dox-dependent *Cyp17* in TCs of the ovary. Following long-term Dox treatment, *Cyp17* mRNA levels from the ovaries of these TC17 mice revealed a Six to ten-fold increase compared with wild-type mouse ovaries. Consistent with overexpression of *Cyp17*, serum levels of T were significantly elevated with no significant change in E2, FSH, or LH. The endocrine profile of our model was also accompanied by significantly greater body and ovarian weight at the end of the treatment compared with controls. In addition, TC17 mice exhibited irregular estrous cycles and were characterized by reduced fertility, with a longer time to first litter and fewer pups per litter than wild-type mice. TC17 morphological ovarian assessment denoted partially impaired folliculogenesis with a significant decrease of antral follicles and hypertrophic stromal cells and increased presence of luteinized stromal cells. We also found high numbers of atretic/cystic follicles and collapsed lucent cell clusters. Collectively, these data suggest an androgen-induced defect in normal folliculogenesis and fertility.

Ovarian morphological features similar to those demonstrated in our TC17 model have been described in prior studies of Testosterone Replacement Therapy (TRT)-treated transgender men [43, 64–68]. Indeed, the TC17 mouse model appeared to resemble specifically several of these features: morphological ovarian assessment in denoted partially impaired folliculogenesis with a significant decrease of antral follicles. Moreover, hypertrophic stromal cells or luteinized stromal cells [69] similar to the ones observed in transgender man ovaries were detected [41, 42, 70, 71]. Although we did not find polycystic ovarian morphology as described by Ikeda et al. we did observe high numbers of atretic/cystic follicles and collapsed lucent cell clusters described by the group [67].

To date, only one animal model has been proposed to investigate the impact of testosterone therapy on reproduction in transgender men. This model, by Kinnear et al*.* utilized subcutaneous administration of testosterone enanthate and mirrored several reproductive perturbations observed in transgender men on T therapy [43, 72]. Interestingly, they showed that T therapy-induced interruption of estrous cyclicity is reversible [72]. However, pregnancy outcomes were not reported for this model, and did not demonstrate the ovarian hypertrophic stromal morphologies observed in humans.

Underlying the morphological changes induced by *Cyp17* overexpression in our TC17 model were several molecular alterations. We found 1011 differentially expressed genes (290 down- and 721 upregulated) in ovaries from TC17 mice compared to those from CTRL mice. Among them, we found genes that can shed light on the ovarian histopathology we described. In the TC17 transcriptomic profile, genes controlling steroid synthesis (*Star*, *Cyp11a1*) were upregulated in the TC17 mice. The LH receptor gene (*Lhcgr*) was also significantly upregulated, explaining the high level of luteinized stromal cells. GO and KEGG analysis of these DEGs corroborated our hypothesis that TC17 can resemble the ovarian phenotype of testosterone-treated transgender men with enrichment of pathways for collagenization and the ECM organization.

Other important evidence of the TGM ovarian phenotype from our transcriptomic data included upregulation of the prolactin receptor (*Prlr*) gene and downregulation of the *Runx1* and *Foxl2* genes. The current literature indicates *Prlr* in the ovary has a luteotropic action [73]. Interestingly, Nicol et al*.* in 2019 found *Runx1* essential for the maintenance of the ovary and the combined loss of *Runx1* and *Foxl2* partially masculinizes fetal ovaries [74]. TC17 was also characterized by polycythemia. High levels of HCT and RBCs are typically increased in TGM, and the subsequent polycythemia is considered an adverse drug reaction lifelong hormonal therapy [75, 76].

Finally, in addition to the described molecular and morphological changes observed in the TC17 mice, impaired fertility was also observed. Our study uncovered that TC17 estrous cycles were disrupted, and pregnancy rates were significantly diminished. This is of particular importance given the lack of clinical data in humans describing the reproductive effects of gender-affirming TRT in TGM. Although TRT is the mainstay of gender-affirming medical care in TGM [77, 78] and secondary amenorrhea is common in testosterone-treated individuals [79–81], the exact mechanism of menstrual suppression is unknown. Although one recent study observed high rates of anovulation in TGM undergoing TRT [82], few studies have assessed the effects of testosterone on ovarian follicle structure and function. Given this limited knowledge, the current standard of care is to counsel patients interested in gender-affirming testosterone therapy after female sex assignment at birth regarding the potential for decreased fertility [77, 78, 83–86]. As the access to gender-affirming care improves, more patients are considering fertility preservation and its impact on their identity and future family goals [87]. As such, there is a critical need to perform a clinical investigation to carefully examine the effects of androgen therapy on normal ovulatory function.

Given the ethical difficulties of studying the reproductive consequences of high-dose testosterone therapy in humans, and the limitations imposed by cost, fecundity, generation time, and lifespan introduced when studying non-human primates, rodent models offer an attractive alternative.

**Fig. 8:**
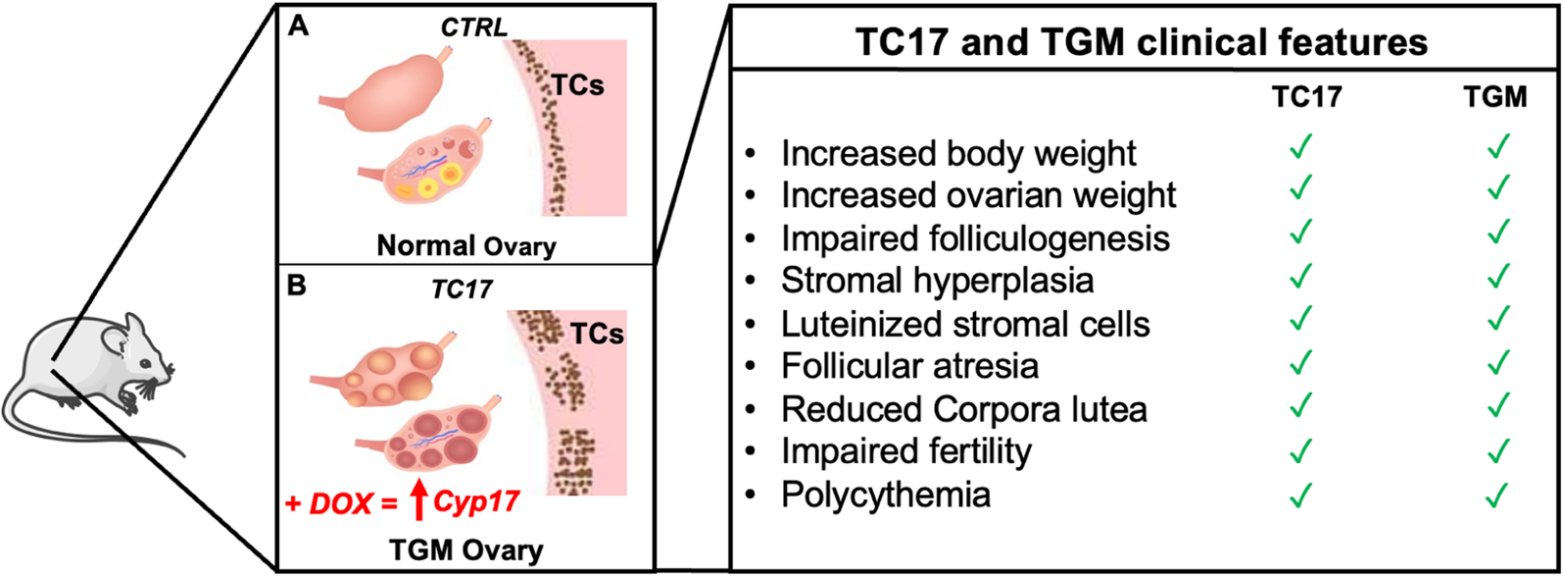
Graphical summary and table of the results. **A** According to our investigation, CTRL mouse ovaries express normal levels of Cyp17 with/without treatment with Doxycycline. **B** Dox administration induces overexpression of *Cyp17* in TC17

Considering that, our investigations show that the novel TC17 model is an innovative and powerful tool for future investigations of the dose-dependent effects of androgen on ovarian structure and function, reproductive cyclicity, and fertility. In summary, TC17: (i) has a doxycycline-dependent regulation Cyp17 specifically in TCs, (ii) resembles TGM ovarian histopathology, (iii) mimics polycythemia condition which is typical in presence of hyperandrogenism (Fig. 8).

## Supplementary Information

**Additional file 1: Figure S1. Doxycycline dose response of Cyp17 expression in TC17 model. **qPCR quantification of the** Cyp17** mRNA expression in CTRL mouse and TC17 mouse ovaries (N=3) respectively treated with 200 mg/Kg Doxycycline for CTRL and 20mg/Kg, 100 mg/Kg, and 200 mg/Kg Doxycycline for TC17 (7 days, i.p. injection every other day). Mean +/- s.e.m. of mouse Cyp17 expression relative to GAPDH housekeeping gene. (**P=0.01), ANOVA. **Figure S2. TC17 ovaries express rtTA/EGFP transactivator. **After Dox treatment (2 weeks)**, **WT and TC17 mice were sacrificed, PFA perfused, and ovaries were collected (N=3). Immunostaining was performed with Cyp17 antibody and Draq5 to stain DNA. Representative confocal micrographs of WT (upper panel) and TC17 ovaries (lower panel). Panels show the effects of Dox treatment in the Cyp17 expression (left, red) and the rtTA/EGFP (middle, green) constitutive expression in TC17 mouse Theca cells. Immunofluorescence co-localization (right, yellow) in the follicles shows the increase in co-expression following exposure to Dox. **Figure S3. Top 50 differentially expressed genes. **Heatmaps of top 50 differentially induced or repressed genes by Cyp17 upregulation found by RNA-seq. (A) Heatmap indicating the top 50 genes upregulated upon *Cyp17* induction (ranked by p-value, adj. p-value < 0.05). (B) Heatmap indicating the top 50 genes downregulated upon Cyp17 induction. **Figure S4. Gene Ontology enrichment. **List of significant GO Terms (biological function) among the total of 1011 differential expressed genes (DEGs) regulated upon *Cyp17* induction. **Figure S5. RNAseq data annotated with Kyoto Encyclopedia of Genes and Genomes (KEGG). **KEGG enrichment analysis in the subsets of upregulated (A) and downregulated (B) of DEGs between TC17 ovaries and CTRL ovaries (N=3). **Figure S6. Molecular analysis of the ovarian markers. **Graphs show fold change means +/- s.e.m relative expression to CTRL following normalization to the housekeeping gene for *Cyp19* (A), *Pgr* (B), *Ahm* (C) and, *Foxl2* (D). Data were analyzed using the two-tailed unpaired t-test (*p<0.05). **Figure S7. Polycythemia in TC17 mice. **Red Blood Cells (RBC, M/μl) and Hematocrit (HMT, %) from CTRL and TC17 were quantified with Hemavet 950FS (N=6/7). Control range for RBC and for HCT (%) were respectively 6.36 M/μl - 9.42 M/μl and 35.1% - 45.4%, means +/- s.e.m. Data were analyzed using the two-tailed Mann-Whitney test (**p<0.01, ***p<0.001).

**Additional file 2: Table S1.**

## Data Availability

Raw sequencing data can be found at the National Center for Biotechnology Information Sequence Read Archive (accession number PRJNA769200).
