# Supplementary material for: Effect of the spatial–temporal specific theca cell Cyp17 overexpression on the reproductive phenotype of the novel TC17 mouse
Source: J Transl Med. 2021 Oct 15;19:428. doi: 10.1186/s12967-021-03103-x (PMC8520195; doi:10.1186/s12967-021-03103-x)
Supplement: Supplementary file 1 — Additional file 1: Figure S1. Doxycycline dose response of Cyp17 expression in TC17 model. qPCR quantification of the Cyp17 mRNA expression in CTRL mouse and TC17 mouse ovaries (N=3) respectively treated with 200 mg/Kg Doxycycline for CTRL and 20mg/Kg, 100 mg/Kg, and 200 mg/Kg Doxycycline for TC17 (7 days, i.p. injection every other day). Mean +/- s.e.m. of mouse Cyp17 expression relative to GAPDH housekeeping gene. (**P=0.01), ANOVA. Figure S2. TC17 ovaries express rtTA/EGFP transactivator. After Dox treatment (2 weeks), WT and TC17 mice were sacrificed, PFA perfused, and ovaries were collected (N=3). Immunostaining was performed with Cyp17 antibody and Draq5 to stain DNA. Representative confocal micrographs of WT (upper panel) and TC17 ovaries (lower panel). Panels show the effects of Dox treatment in the Cyp17 expression (left, red) and the rtTA/EGFP (middle, green) constitutive expression in TC17 mouse Theca cells. Immunofluorescence co-localization (right, yellow) in the follicles shows the increase in co-expression following exposure to Dox. Figure S3. Top 50 differentially expressed genes. Heatmaps of top 50 differentially induced or repressed genes by Cyp17 upregulation found by RNA-seq. (A) Heatmap indicating the top 50 genes upregulated upon Cyp17 induction (ranked by p-value, adj. p-value < 0.05). (B) Heatmap indicating the top 50 genes downregulated upon Cyp17 induction. Figure S4. Gene Ontology enrichment. List of significant GO Terms (biological function) among the total of 1011 differential expressed genes (DEGs) regulated upon Cyp17 induction. Figure S5. RNAseq data annotated with Kyoto Encyclopedia of Genes and Genomes (KEGG). KEGG enrichment analysis in the subsets of upregulated (A) and downregulated (B) of DEGs between TC17 ovaries and CTRL ovaries (N=3). Figure S6. Molecular analysis of the ovarian markers. Graphs show fold change means +/- s.e.m relative expression to CTRL following normalization to the housekeeping gene for Cyp19 (A), Pgr (B), [file 12967_2021_3103_MOESM1_ESM.docx]

**Additional file 1:**

**
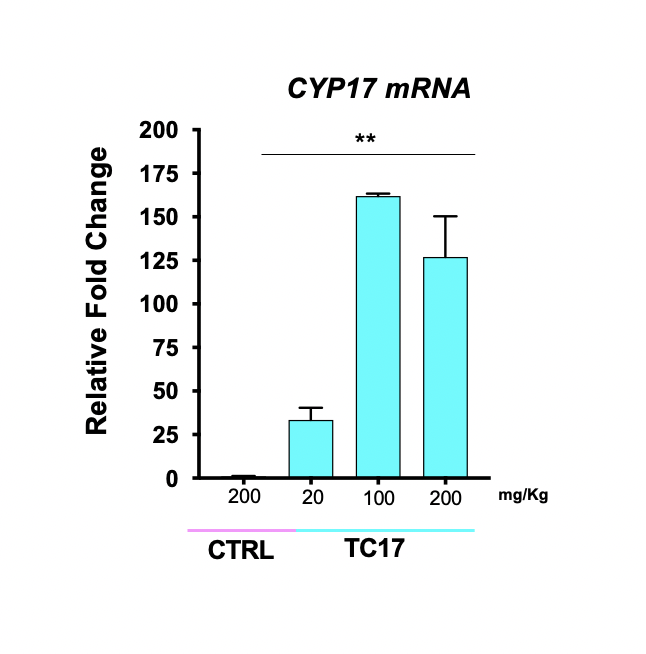
**

**Figure S1. Doxycycline dose response of Cyp17 expression in TC17 model.**

qPCR quantification of the *Cyp17* mRNA expression in CTRL mouse and TC17 mouse ovaries (N=3) respectively treated with 200 mg/Kg Doxycycline for CTRL and 20mg/Kg, 100 mg/Kg, and 200 mg/Kg Doxycycline for TC17 (7 days, i.p. injection every other day). Mean +/- s.e.m. of mouse Cyp17 expression relative to GAPDH housekeeping gene. (**P=0.01), ANOVA.


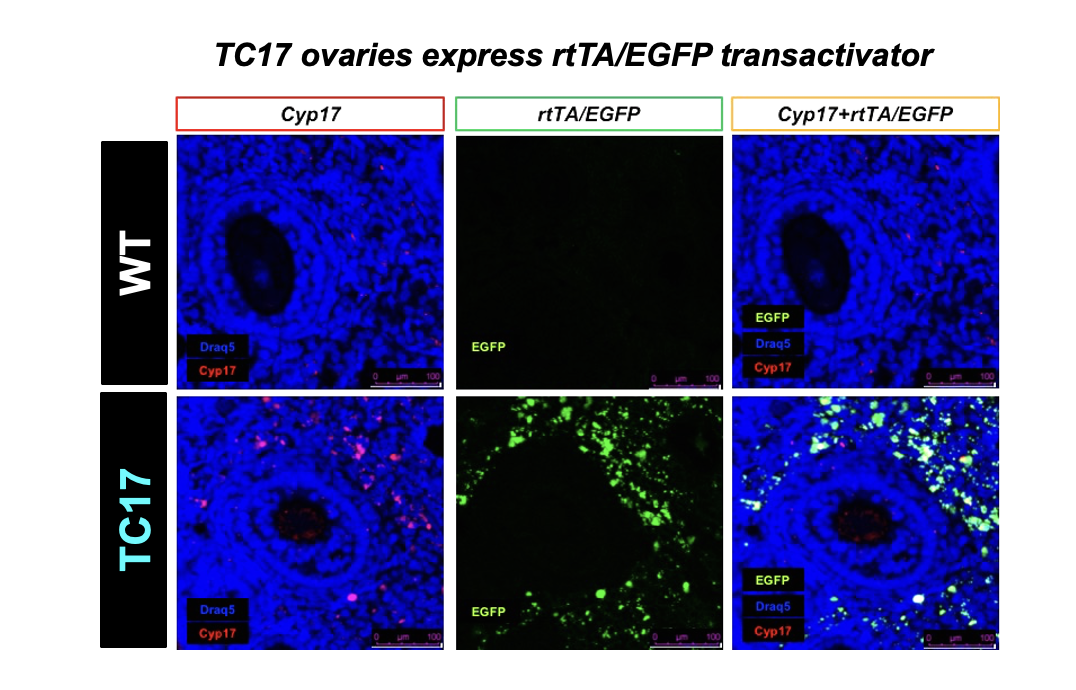


**Figure S2. TC17 ovaries express rtTA/EGFP transactivator.** After Dox treatment (2 weeks)**,** WT and TC17 mice were sacrificed, PFA perfused, and ovaries were collected (N=3). Immunostaining was performed with Cyp17 antibody and Draq5 to stain DNA. Representative confocal micrographs of WT (upper panel) and TC17 ovaries (lower panel). Panels show the effects of Dox treatment in the Cyp17 expression (left, red) and the rtTA/EGFP (middle, green) constitutive expression in TC17 mouse Theca cells. Immunofluorescence co-localization (right, yellow) in the follicles shows the increase in co-expression following exposure to Dox.

**
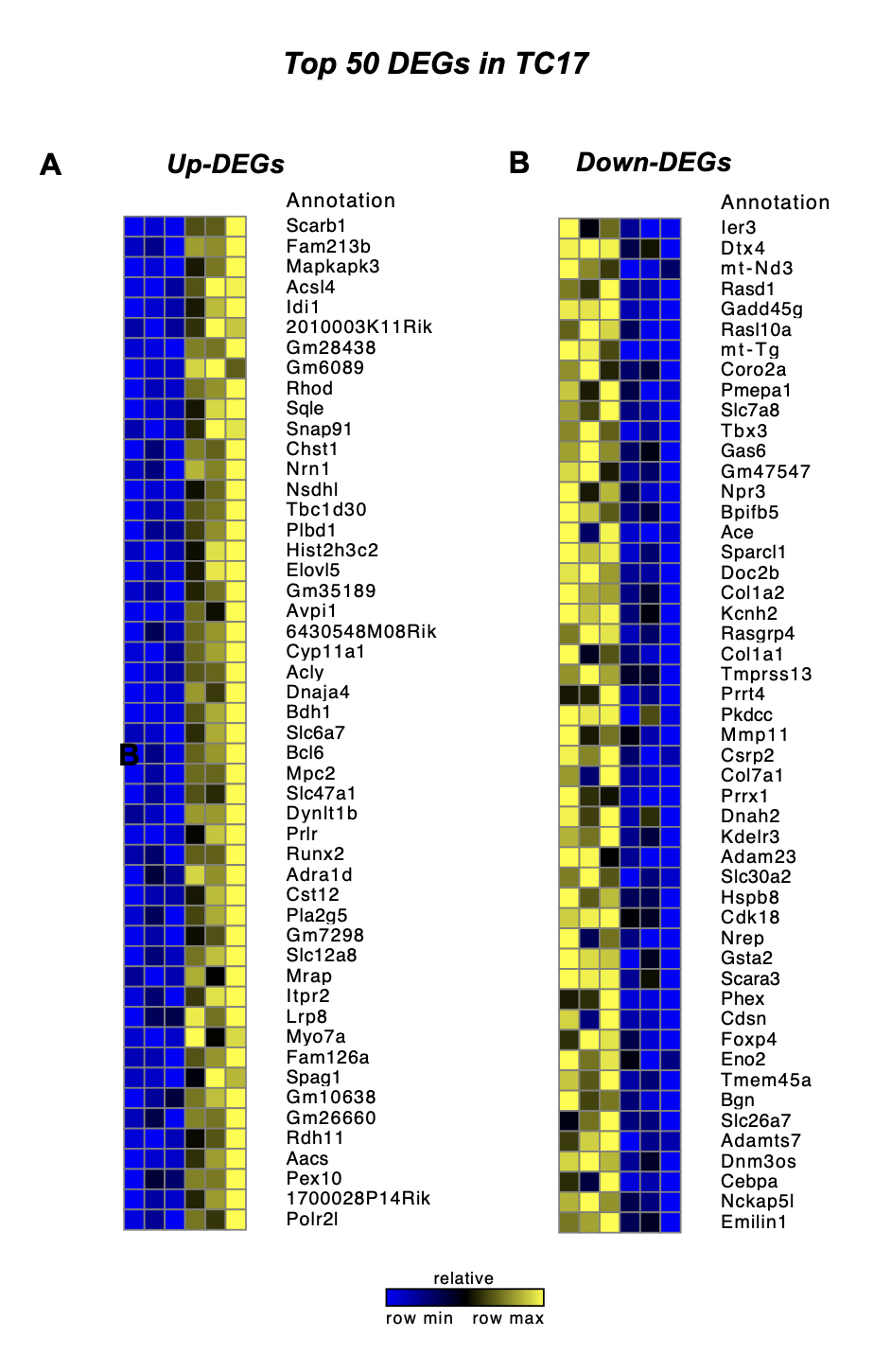
**

**Figure S3. Top 50 differentially expressed genes.** Heatmaps of top 50 differentially induced or repressed genes by Cyp17 upregulation found by RNA-seq. (A) Heatmap indicating the top 50 genes upregulated upon *Cyp17* induction (ranked by p-value, adj. p-value < 0.05). (B) Heatmap indicating the top 50 genes downregulated upon Cyp17 induction.

**
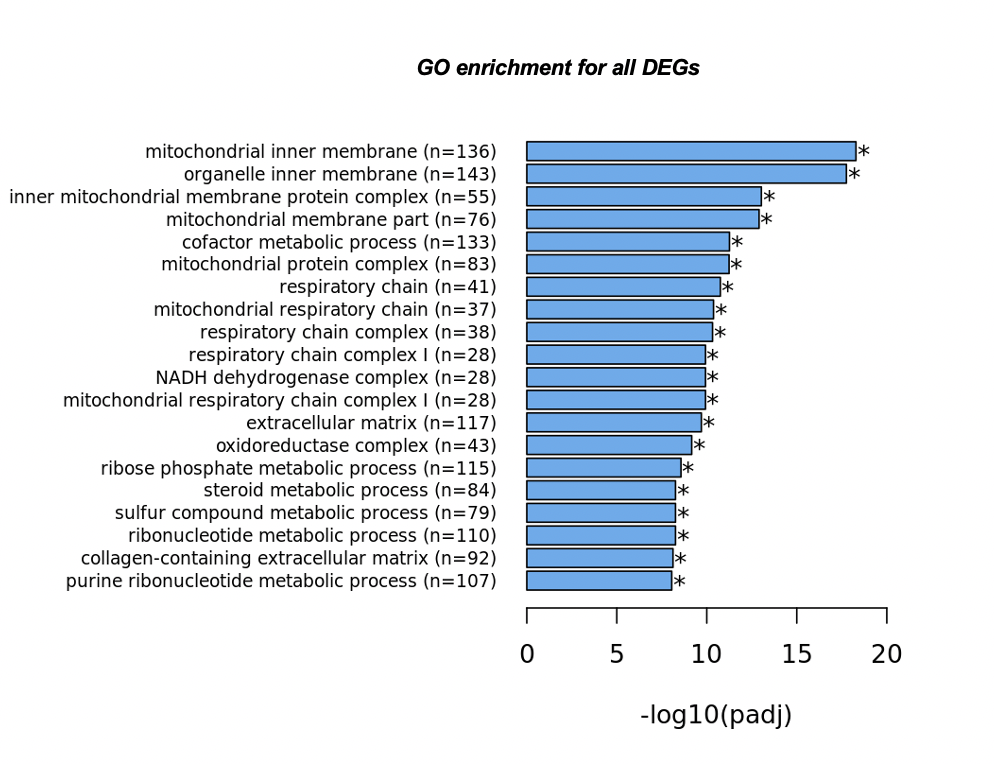
**

**Figure S4. Gene Ontology enrichment.** List of significant GO Terms (biological function) among the total of 1011 differential expressed genes (DEGs) regulated upon *Cyp17* induction.

**
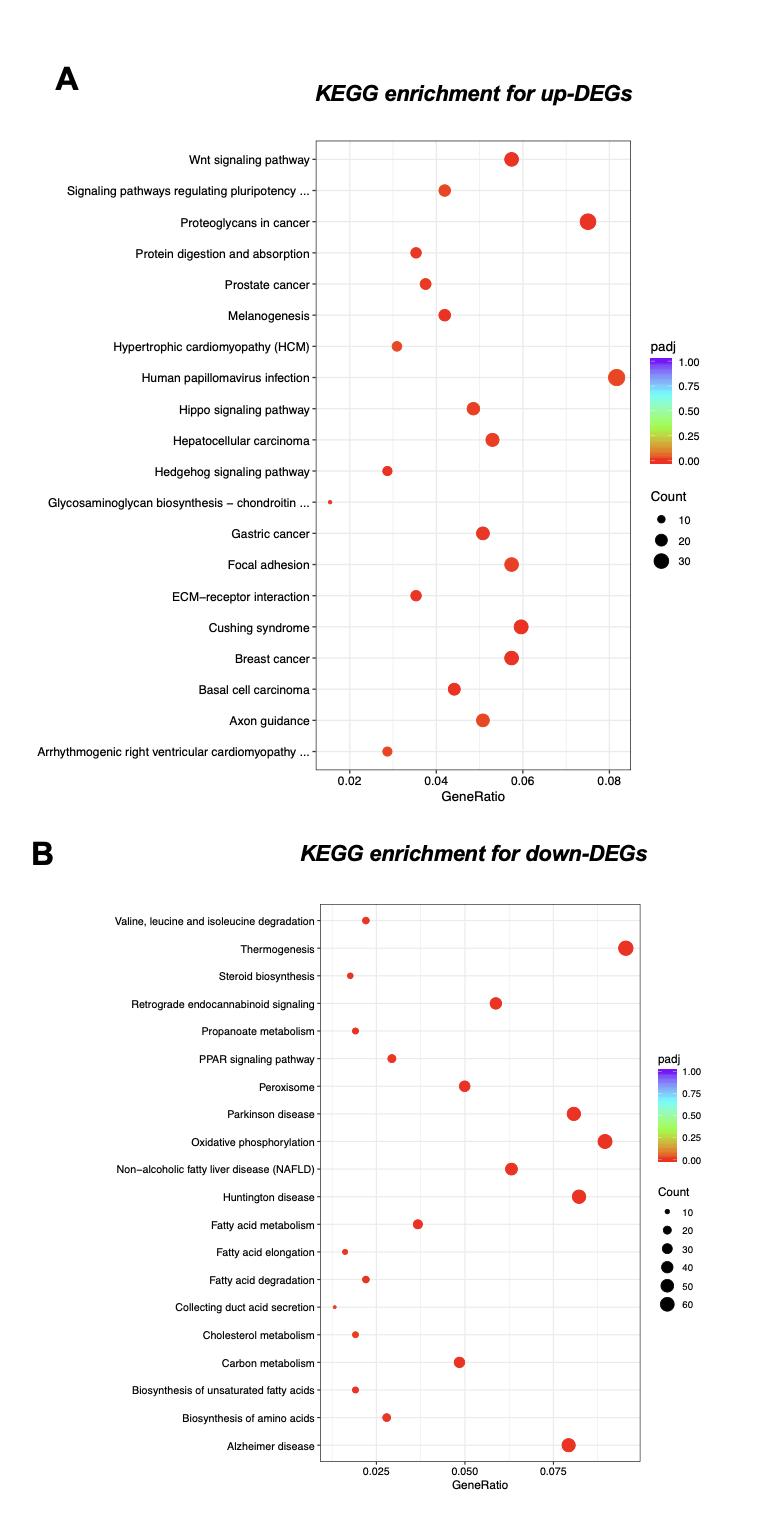
**

**Figure S5. RNAseq data annotated with Kyoto Encyclopedia of Genes and Genomes (KEGG).** KEGG enrichment analysis in the subsets of upregulated (A) and downregulated (B) of DEGs between TC17 ovaries and CTRL ovaries (N=3).


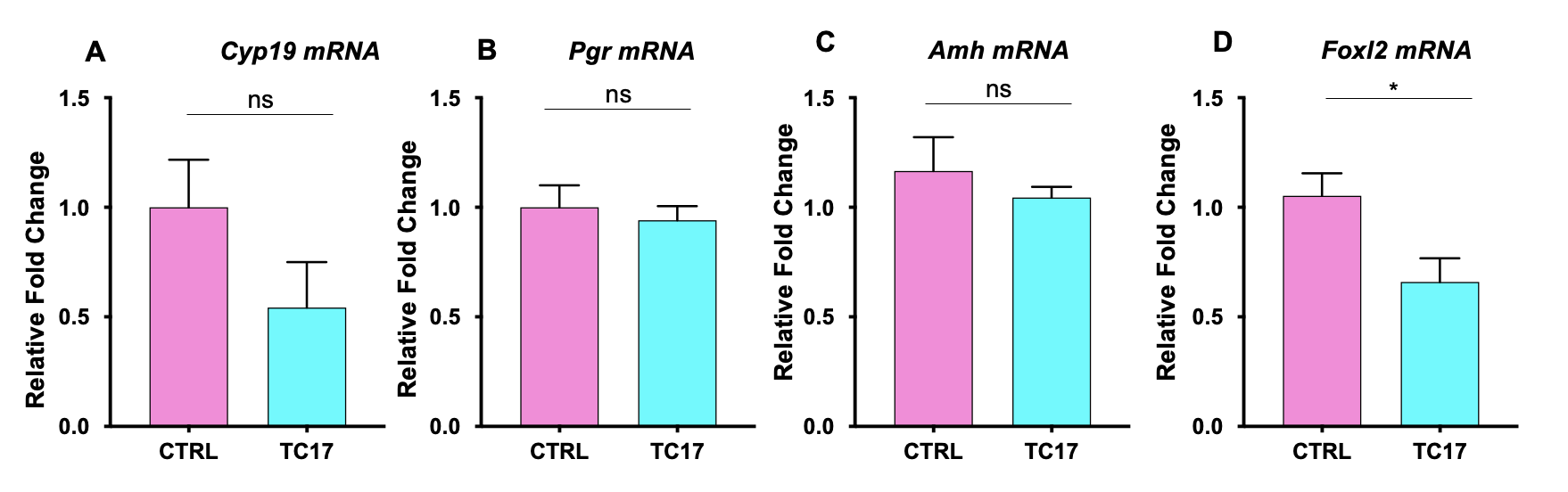


**Figure S6. Molecular analysis of the ovarian markers.** Graphs show fold change means +/- s.e.m relative expression to CTRL following normalization to the housekeeping gene for *Cyp19* (A), *Pgr* (B), *Ahm* (C) and, *Foxl2* (D). Data were analyzed using the two-tailed unpaired t-test (*p<0.05).

**
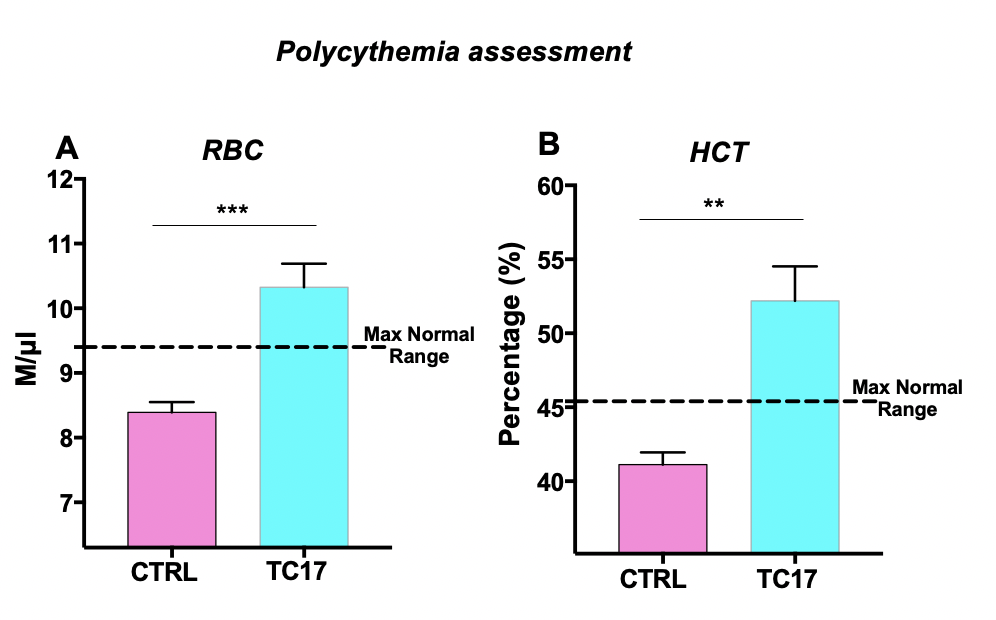
**

**Figure S7. Polycythemia in TC17 mice.** Red Blood Cells (RBC, M/μl) and Hematocrit (HMT, %) from CTRL and TC17 were quantified with Hemavet 950FS (N=6/7). Control range for RBC and for HCT (%) were respectively 6.36 M/μl - 9.42 M/μl and 35.1% - 45.4%, means +/- s.e.m. Data were analyzed using the two-tailed Mann-Whitney test (**p<0.01, ***p<0.001).
